# Microbial degradation of terrigenous dissolved organic matter and potential consequences for carbon cycling in brown-water streams

**DOI:** 10.1038/srep04981

**Published:** 2014-05-15

**Authors:** Christina Fasching, Barbara Behounek, Gabriel A. Singer, Tom J. Battin

**Affiliations:** 1 Department of Limnology and Oceanography, University of Vienna, Althanstrasse 14, A-1090 Vienna, Austria; 2 WasserCluster Lunz GmbH, Dr. Carl Kupelwieser Promenade 5, 3293 Lunz am See, Austria; 3 Current address: Department of Ecohydrology, Leibniz-Institute of Freshwater Ecology and Inland Fisheries (IGB), Müggelseedamm 310, 12587 Berlin, Germany.

## Abstract

Streams receive substantial terrestrial deliveries of dissolved organic matter (DOM). The chromophoric (CDOM) fraction of terrestrial deliveries confers the brown colour to streamwater, often understood as browning, and plays a central role in aquatic photochemistry and is generally considered resistant to microbial metabolism. To assess the relevance of terrigenous DOM for carbon fluxes mediated by stream microorganisms, we determined the bioavailable fraction of DOM and microbial carbon use efficiency (CUE), and related these measures to partial pressure of CO_2_ in headwater streams spanning across a browning gradient. Fluorescence and absorbance analyses revealed high molecular weight and aromaticity, and elevated contributions from humic-like components to characterize terrestrial CDOM. We found that microorganisms metabolized this material at the cost of low CUE and shifted its composition (from fluorescence and absorbance) towards less aromatic and low-molecular weight compounds. Respiration (from CUE) was related to CO_2_ supersaturation in streams and this relationship was modulated by DOM composition. Our findings imply that terrigenous DOM is respired by microorganisms rather than incorporated into their biomass, and that this channelizes terrigenous carbon to the pool of CO_2_ potentially outgassing from streams into the atmosphere. This finding may gain relevance as major terrigenous carbon stores become mobilized and browning progresses.

A significant amount of the terrestrial primary production is transported laterally along the fluvial continuum from soils to the oceans^1^. Evidence suggests that this lateral flux as DOM into inland waters is increasing in North America, North and Central Europe, and in Southeast Asia^1^,^2^,^3^,^4^,^5^ causing visible browning of these waters^6^. Especially the CDOM fraction of these terrestrial deliveries impacts on freshwater ecosystems as CDOM influences the light regime, primary production and related trophic processes^4^,^7^. At high concentration, CDOM can even have implications for drinking water supply^8^. The photochemical degradation of CDOM can also contribute to CO_2_ outgassing fluxes from streams, rivers and lakes^9^.

It is therefore of paramount relevance to improve our understanding of the carbon cycling at the terrestrial-aquatic interface^10^,^11^,^12^. This is especially true for headwater streams, which are most abundant in fluvial networks and tightly connected with the terrestrial milieu. Small streams are also major sources of CO_2_ to the atmosphere^10^,^13^,^14^ but the contributions of DOM metabolism to CO_2_ outgassing from these streams remain poorly understood. The common wisdom that terrestrial CDOM (that is, mostly humics) is resistant^15^ to microbial metabolism has shaped our thinking over the last decades^12^. This perception is now being re-evaluated based on evidence that chemical recalcitrance and high Δ^14^C age as commonly assumed properties of terrigenous DOM do not necessarily predict its bioavailability^12^,^16^,^17^. It was shown for instance that terrestrial DOM appears to drive respiration and CO_2_ outgassing from the Amazon River^17^,^18^.

The aim of this study was to investigate the metabolic fate of terrestrial DOM in headwater streams and to assess its potential contribution to carbon cycling in these streams. Study streams encompassed a brown-colour gradient and we used a space-for-time substitution approach to evaluate the effect of browning on carbon dynamics. We hypothesize that heterotrophic metabolism in stream ecosystems, as depicted by *p*CO_2_, results from dissolved organic carbon (DOC) concentration, its bioavailable fraction (%BDOC) and the catabolic response of the microorganisms as carbon use efficiency (CUE). We assume that %BDOC and CUE both depend on DOM composition^19^,^20^. To test this hypothesis, we related DOC concentration and DOM optical properties (from fluorescence spectrometry and absorbance) to %BDOC, CUE and to the potential evasion of CO_2_ in brown-water streams.

## Results

### DOM quantity and composition

**Figure 1:**
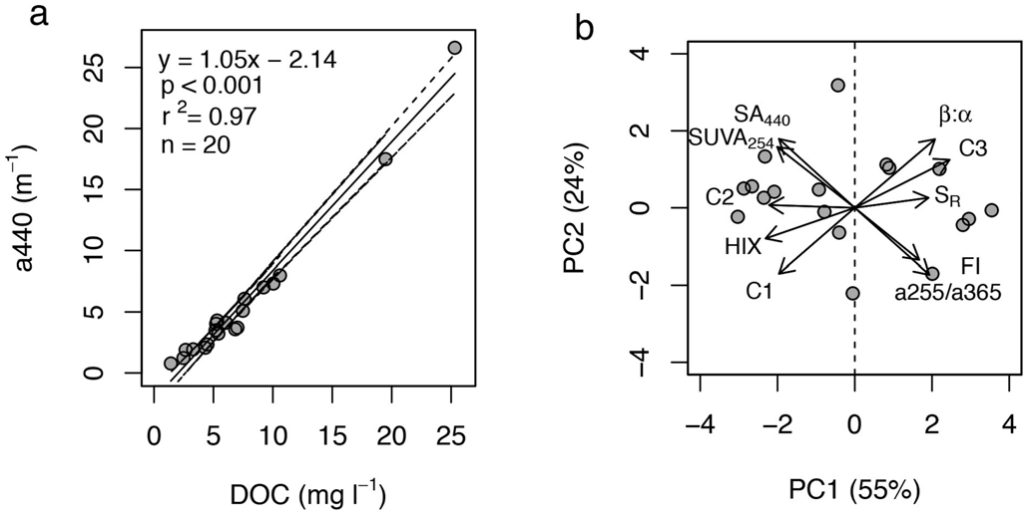
Quantitative and qualitative aspects of DOM. (a) Colour (as *a*_440_) increases with DOC concentration. Dashed lines represent 95% confidence intervals. (b) Principal component analysis (PCA) based on the optical properties of the 20 streams distinguishes terrigenous from autochthonous DOM (PC1). Terrigenous DOM was characterized by high specific absorption at 254 nm (SUVA_254_) and 440 nm (SA_440_), and a high humification index (HIX). Fluorometry revealed two humic-like compounds of terrigenous origin (C1 and C2), while the third component (C3) was putatively associated with new biological production (Supplementary Information). High values of the freshness index (β/α) and the fluorescence index (FI) but also lower apparent molecular weight (indicated by a255/a365 and the slope ratio S_R_) describe DOM with a more autochthonous character. Arrows are based on PCA structural coefficients.

We investigated attributes of DOM from twenty 1^st^-order streams in Austria (Table S1 and Table S2) that drain catchments spanning over a gradient in brown colour as a response of varying in peat cover. In fact, the relative contributions of peat and wetlands to land cover are known to imprint on CDOM and its colour as a commonly used indicator of terrestrial DOM deliveries to freshwater ecosystems^21^,^22^,^23^,^24^. We found streamwater DOC concentration ranging from 1.41 to 24.31 mg C L^−1^ and CDOM colour (*a*_440_) ranging from 0.78 to 26.64 m^−1^ (Table S1) across all streams; concentration and colour correlated significantly (Figure 1a).

**Figure 2:**
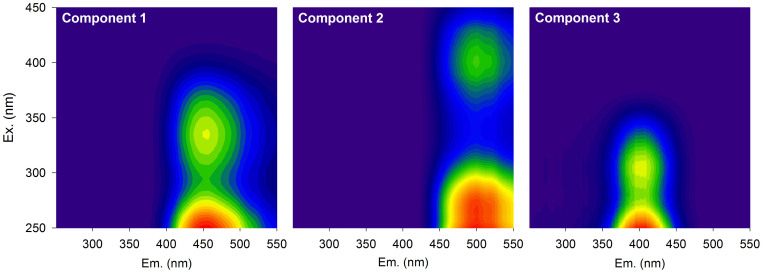
Three fluorescent components were modeled by parallel factor analysis (PARAFAC) from excitation emission matrices. All components were assigned as humic-like.

We analysed the composition of streamwater DOM as inferred from absorbance and fluorescence measures including fluorescent components modelled from excitation emission matrices (EEMs) by parallel factor analysis (PARAFAC)^25^. A principal component analysis (PCA) based on these measures revealed a gradient of streams where terrigenous DOM mixes in varying proportions with autochthonous DOM. Elevated aromaticity (SUVA_254_), colour (*a*_440_), higher degree of humification (HIX) and apparent molecular-weight compounds (S_R_), and the humic-like fluorescent components C1 and C2 (Figure 2) are all indicative of terrigenous DOM deliveries to these streams. On the other hand, the freshness index (β/α), low apparent molecular weight and aromaticity, but also the humic-like component C3 (Figure 2) assumedly associated with biological production^26^, indicate in-stream production of DOM.

**Figure 3:**
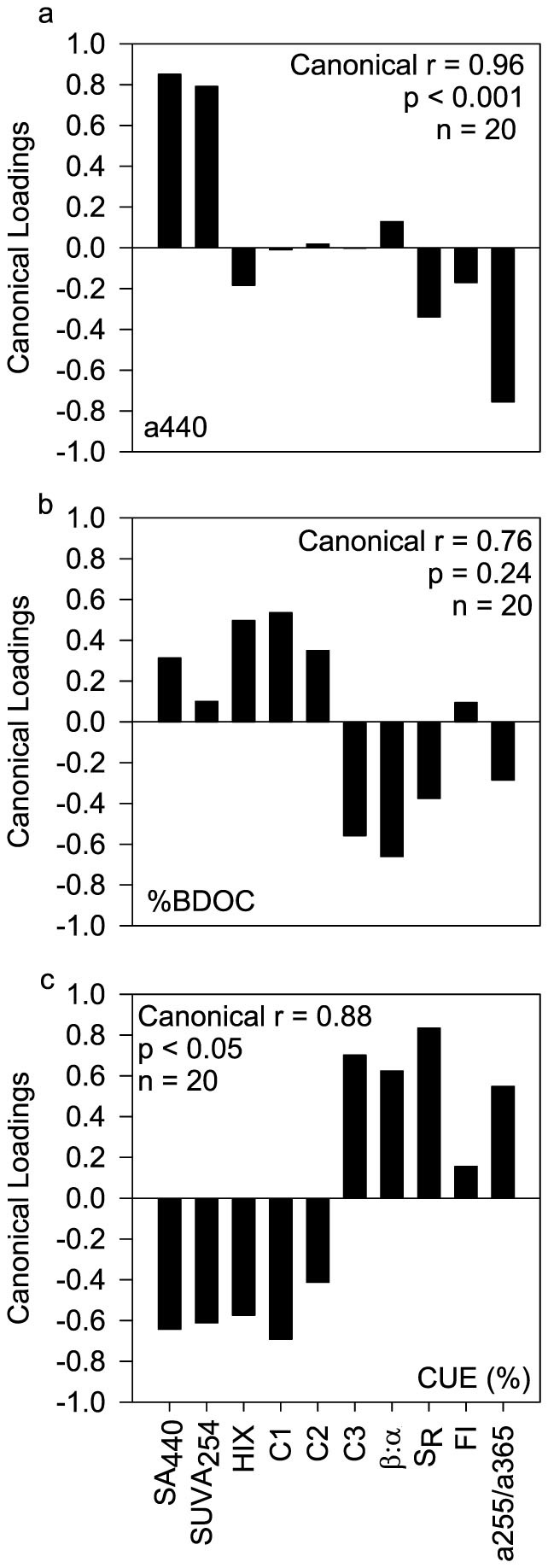
DOM composition is related to colour (*a*_440_) and impacts microbial metabolism. Canonical correlation analyses reveal DOM optical properties most strongly related to (a) colour (*a*_440_), (b) the bioavailable fraction of DOC (%BDOC) and (c) carbon use efficiency (CUE). Canonical loadings (correlations between the one canonical variate of our analysis and the respective variables) indicate the strength and direction of the relationship between the respective constraint and individual optical measures (*see Methods*).

Relating the same set of concentration-independent optical measures that describe DOM composition to *a*_440_, we found that the terrigenous imprint on DOM increases with brown colour across all streams (Figure 3a). To rule out the possibility of a spurious correlation driven by DOC concentration, we excluded SA_440_ and SUVA_254_ from the analysis and were able to confirm the observed composition shift towards terrigenous DOM paralleled the gradient of brown colour (Figure S1).

### DOM biodegradation and CUE

We used classical batch biodegradation assays (20 days) to monitor changes in DOM composition upon microbial degradation and calculated BDOC (as percent of the initial DOC concentration)^27^ and CUE (as biomass production *versus* biomass production plus respiration). Based on canonical correlations, we did not find any obvious relationship between %BDOC and DOM composition as inferred from its optical properties (Figure 3b and Figure S2). To back up our BDOC measurements we also computed microbial carbon demand from respiration and biomass build-up^20^, which should essentially equal BDOC. The linear relationship between both measures (r^2^ = 0.80, p < 0.001, n = 20) (see Supplementary Information) supports our BDOC measurements.

In contrast to %BDOC, CUE varied more broadly (4.57% to 37.10%) over all twenty study streams and averaged 12.73 ± 9.58%; CUE was significantly related to DOM composition (canonical correlation, r = 0.88, p < 0.05, n = 20). CUE increased with putatively fresh DOM of apparently low molecular weight and with a microbial imprint; it declined with increasing contributions from terrigenous DOM (Figure 3c and Figure S3). To test whether coloured DOM affects CUE, we also related the gradient of DOM quality associated with CUE to the gradient of DOM quality associated with brown colour and found a significant correlation (Spearman correlation, r = −0.50, p < 0.05).

**Figure 4:**
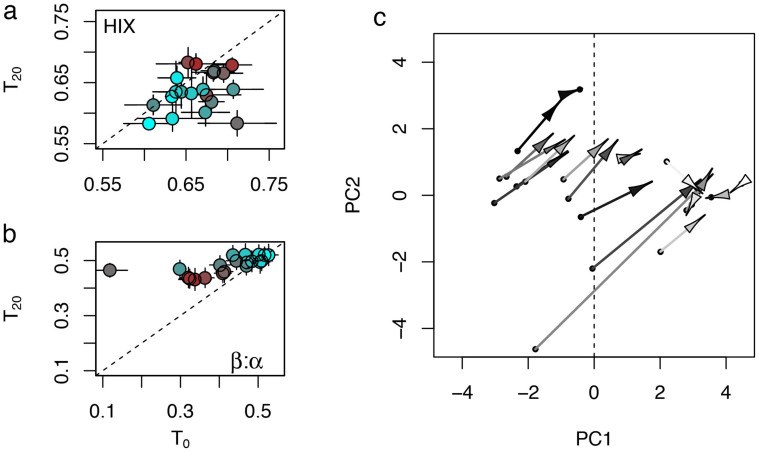
DOM composition during microbial degradation. (a) The humification index (HIX) and (b) the freshness index (β/α) at start of the degradation assays (T_0_) and after the 20-day incubation period (T_20_). Given is the mean ± SD (n = 3 replicates) and the dashed line indicates a 1:1 relationship (i.e., no change during degradation) Original DOM composition as derived from the PCA is indicated by colour. Brown colours denote streams with predominantly terrigenous DOM and blue colours represent streams with relatively more autochthonous DOM. (c) Shifts in DOM composition over the 20-day experiments. Starting coordinates of arrows (indicated by black circles) are identical with scores of the PCA based on optical measures (Figure 1b). End coordinates are predicted scores computed with the PCA eigenvectors and the optical measures at the end of the experiments. Lengths of arrows denote the intensity of the shift in DOM composition. The greyscale indicates CUE; ranging from black (lowest CUE) to white (highest CUE).

**Figure 5:**
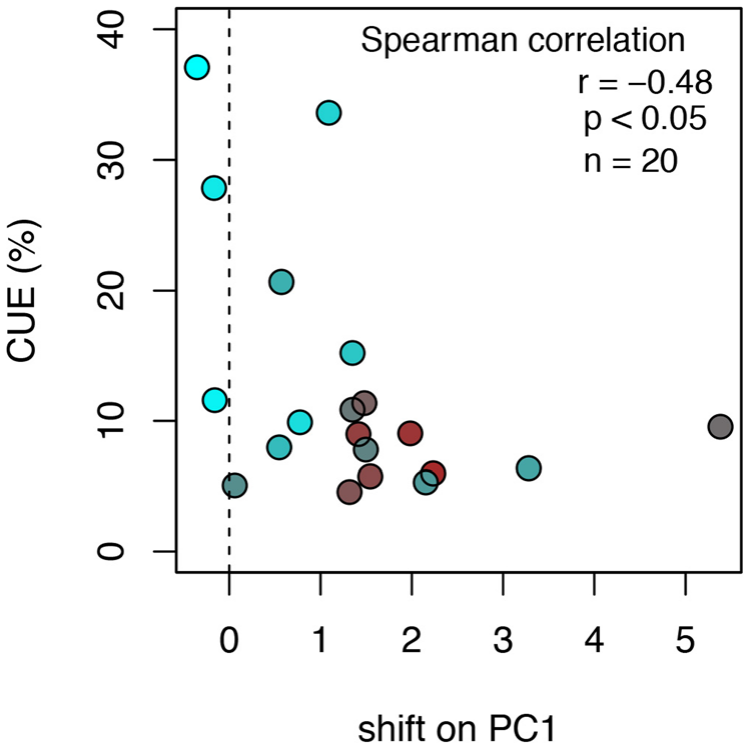
CUE decreases with the absolute shift in DOM composition along PC1 (i.e., each stream's PC1 score *after* minus the respective score *before* degradation). Original DOM composition as derived from the PCA (PC1) is indicated by colour. Brown colour denotes streams with predominantly terrigenous DOM and blue colour represents streams with relatively more autochthonous DOM.

DOM optical properties shifted upon microbial degradation as indicated from a decrease of humification (as HIX) (t-test, p < 0.001, n = 20; Figure 4a) and a concurrent increase of the apparent freshness (as β/α) of DOM (t-test, p < 0.001, n = 20; Figure 4b). Especially when the initial terrigenous imprint was strong, apparent freshness increased disproportionally upon degradation. We depicted these shifts in DOM composition occurring during the degradation assays by using the eigenvectors of the PCA describing DOM composition (based on the optical information across the 20 streams before running biodegradation assays) to predict PCA-scores at the end of the experiments (Figure 4c and Figure S4). This revealed that the initial terrigenous imprint on DOM decreased upon microbial degradation, which resulted in an enhanced autochthonous DOM signature. Relating the length of these shifts in carbon composition to CUE, we found that that DOM with a stronger terrestrial imprint induced lower CUE (Figure 5).

### Relationship between BDOC, CUE and streamwater CO_2_

To evaluate carbon cycling in our study streams we first determined CO_2_ partial pressure (*p*CO_2_) in the streamwater and found an average *p*CO_2_ of 1,611 ± 1,055 μatm. We also calculated the degree of CO_2_ supersaturation (expressed as ΔCO_2_, which is the concentration of streamwater CO_2_ minus the CO_2_ concentration expected at complete atmospheric equilibrium) as a proxy for stream ecosystem metabolism. Positive ΔCO_2_ values (73.00 ± 64.48 μmol l^−1^) indicate that all streams were supersaturated in CO_2_ and hence a potential source of CO_2_ to the atmosphere. Next we related respiration rates measured in the biodegradation assays and found them closely related to ΔCO_2_ in the streams (r^2^ = 0.66, p < 0.001, n = 20). To further illuminate the processes driving this relationship, we propose the following linear model 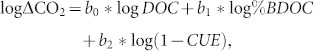 which is derived from our understanding that stream metabolism is the product of the total amount of DOC *times* its bioavailable fraction (%BDOC) *times* the catabolic response of the microorganisms, as the fraction respired carbon (1-CUE). Regression analysis revealed that DOC concentration and CUE explained 51% (p < 0.01) of the variance in ΔCO_2_ across all study sites (Figure S5). Although DOC concentration (beta = 0.45, p < 0.05, sum of squares = 3.91) was the strongest predictor for ΔCO_2_ followed by CUE as suggested by its sums of squares (beta = 0.53, p < 0.05, sum of squares = 1.68). BDOC was not a significant predictor (beta = −0.17, p = 0.43, sum of squares = 0.2).

## Discussion

### DOM quantity and composition

Inland waters receive large amounts of DOM from the terrestrial environment^1^. The traditional perception that the chromophoric and humic fraction of this delivery is largely resistant to the microbial metabolism is now changing^12^,^16^,^17^. Our findings expand this changing view by showing the link between the microbial degradation of terrestrial CDOM and potential CO_2_ outgassing from small headwater streams.

Our results reveal that terrestrial deliveries not only increase the DOC concentration in streams but also shift the composition of DOM. The DOC concentrations we measured are closely bracketed by values from headwaters in the UK^2^ and North America^30^, for instance, and colour (*a*_440_) is in the range of values (0.69–29.94 m^−1^) from boreal streams and rivers^9^. PARAFAC analyses revealed that humic-like compounds and elevated aromaticity (as SUVA_254_) characterised terrigenous DOM. Values of SUVA_254_ from our streams are in the upper range of values reported from streams and lakes in North America^28^ (range: 0.3–8.7 mg L^−1^ m^−1^) and closely bracketed by values (3.5 to 4.9 mg L^−1^ m^−1^) reported from channels draining tropical peat swamp forests^5^. The browning gradient that we captured with our study streams may thus be representative for other systems influenced by increased terrestrial DOM deliveries.

The relationship between DOC concentration and colour indicates that the variability in DOC concentration across the streams is driven to a large extent by terrigenous DOM deliveries^9^,^21^,^29^. Clearly, this relationship supports the quantitative effect of “brown” and terrigenous DOM deliveries on stream DOC concentration as reported previously^2^,^31^. The canonical correlation analysis furthermore revealed that an increase in colour was accompanied by high aromaticity, high apparent molecular weight and high humification collectively pointing at the increasing terrigenous character of DOM. This is also supported by lower FI values indicative of allochthonous DOM sources^32^ and by elevated contributions of the humic-like fluorescence components C1 and C2. Both fluorescence components are of terrestrial origin^33^ and often encountered in freshwater ecosystems^34^, and thus appropriate to track DOM sources^35^. Our findings corroborate previous studies relating DOM colour to chemical composition, including apparent molecular weight^36^ or lignin phenols^37^.

The PCA based on DOM absorbance and fluorescence also suggests that autochthonous compounds imprint to varying degrees on the optical properties of the DOM pool. Such autochthonous DOM compounds may be of recent microbial origin as indicated by elevated values of the freshness index (β/α)^38^,^39^. High values of the fluorescence index (FI) commonly used as an indicator of DOM source (i.e., terrigenous *versus* microbially derived)^32^ along with lower apparent molecular weight and aromaticity further support our notion of microbial contributions to the DOM pool.

The relative mix of allochthonous (that is, terrestrial) and autochthonous DOM across the browning gradient studied here possibly affects the susceptibility of the DOM pool to the microbial metabolism in the streams. We could not find any significant effect of DOM composition on its bioavailability (as %BDOC). This seems to run counter to findings from coastal temperate streams, for instance, where protein-like fluorescence predicted %BDOC ranging on average from 12.6% and 30.1%^40^. We attribute this discrepancy to the comparatively low %BDOC values, but close to those reported from other streams draining peat^41^, and to their low variability (average ± SD: 4.55 ± 2.14%) across our study streams. CUE, however, varied broadly across the streams and was related to DOM composition. This agrees with previous findings^19^, showing, for instance, a relationship between CUE and apparent molecular weight (as *a*254/*a*365)^42^. In line with this, CUE increased with putatively fresh DOM of apparently low molecular weight and with a microbial imprint; it declined with increasing contributions from terrigenous DOM. CUE was lower than values reported from various aquatic ecosystems^19^, which may be attributable to the largely terrigenous character of DOM as the consumption of terrigenous carbon may support a low but continuous level of metabolic activity^43^. Our data on nutrient concentration in the biodegradation assays (Table S2) preclude that nutrient limitation affected CUE. This is important to note as nutrient limitation can shift the balance between respiration and biomass production when excess carbon is released by respiration^44^. CUE may also be low in the absence of nutrient limitation. For instance, the relative energy content per unit carbon may decrease with increasing terrestrial imprint due to more oxidized compounds^19^,^45^. The degradation of such compounds requires the production of exoenzymes (e.g., phenoloxidases), which increases carbon demand but which may also enhance respiration^45^.

The fact that the gradient of DOM composition associated with CUE paralleled the gradient of DOM composition (that is, CDOM associated with the brown colour) indicates that changes in this CDOM lead to a decrease in CUE. This suggests an impact of brown DOM on carbon processing in streams. In streams with elevated brown colour, a smaller fraction of DOC is incorporated into microbial biomass, while a larger fraction is used for the maintenance of microbial metabolism.

Biodegradation assays revealed that microbial activity apparently imparted an autochthonous signature on an otherwise allochthonous DOM pool characterised by chromophoric and humic-like terrigenous compounds. Our findings suggest that microorganisms removed humic-like compounds while simultaneously producing novel compounds. This is consistent with the perception of the dual role of microorganisms as consumers and producers of DOM^46^. Uptake of humic substances or polyphenolic compounds by microorganisms in streams has been reported previously^43^,^45^,^47^. DOM with a strong initial terrigenous imprint was extensively reworked as indicated by the pronounced shifts towards autochthonous DOM compared to DOM that initially has a more autochthonous signature. The apparent freshness for instance increased disproportionally when the initial terrigenous imprint on DOM was strong, which is reflected in the overall shifts in DOM composition upon degradation. This agrees with the observation of lignin and related phenolic compounds being extensively reworked by microorganisms and possibly causing a complete overturn of the DOM pool within weeks in the Amazon River^17^. Moreover, low-molecular-weight DOM of terrigenous origin was readily available and also quantitatively important for microbial metabolism in boreal freshwaters^20^. However, optical properties of relatively more autochthonous DOM changed comparatively less upon microbial degradation, which may be due to the simultaneous production of novel compounds which may resemble the optical properties of the initial material.

DOM with a strong terrigenous imprint induced lower CUE upon degradation, supporting the results from the canonical correlation analysis. This confirms that this carbon pool fuelled microbial respiration rather than sustaining microbial growth. This unbalance between microbial respiration and growth may impart the microbial imprint on DOM observed during biodegradation. As evoked by the shifts in DOM composition this may occur especially when the initial terrestrial imprint was strong. Our results suggest that elevated catabolism was likely required to maintain microbial metabolism of terrigenous DOM^19^.

It is now well established that headwater streams are supersaturated in CO_2_^9^,^14^,^13^,^48^,^49^,^50^,^51^, yet it remains largely unclear to what extent this CO_2_ originates from catchment or from *in situ* respiration^12^. While there is evidence that groundwater delivers CO_2_ from terrestrial respiration into small headwater streams^49^,^50^,^52^, it is also recognized that these streams are net heterotrophic (that is, they metabolize terrigenous organic carbon)^10^. That inland waters are to a large extent net heterotrophic is also supported by surveys showing that lake and stream *p*CO_2_ is linked to *in situ* metabolism of DOC^9^,^48^,^51^. In line with these observations, the respiration measured in our biodegradation assays was closely related to streamwater ΔCO_2_, which indicates indeed that metabolism contributes to the build-up of CO_2_ in headwater streams. This result is remarkable because it links DOC metabolism to CO_2_ supersaturation in the streams despite the fact that these are independent measurements.

To further explore the relationship between respiration and CO_2_ supersaturation in the streams we proposed a simple model based on the fact that the fraction of respired DOC can be inferred from BDOC and CUE. Our model revealed DOC concentration and CUE as significant predictors for streamwater ΔCO_2_. DOC concentration may affect streamwater ΔCO_2_ on a mass basis, which complies with large-scale surveys from lakes and streams^48^,^51^. We recognise that a common terrestrial origin of both DOC and CO_2_ may drive to some extent at least the observed relationship between DOC and streamwater CO_2_ as reported from Amazonian headwater streams^52^. It is clear that quantitative contributions of DOC and CUE to CO_2_ supersaturation cannot be inferred from our model; however, it points to the microbial metabolism of terrigenous DOM as an important driver of CO_2_ dynamics in brown-water streams. While DOC represents the potential energy basis for the heterotrophic metabolism, BDOC better reflects the amount of organic carbon that is actually available for microbial metabolism. That %BDOC was not a significant term points to bioavailable DOM as a relatively constrained fraction of the carbon pool, which in fact agrees with the missing relationship between %BDOC and DOM composition. In contrast, model results stress that the coupling of CUE with DOM composition constitutes a control on ΔCO_2_ besides DOC quantity. Our findings thus provide evidence that DOM quantity and composition affect CO_2_ build-up in headwater streams. The quantity of terrigenous DOM deliveries to streams may increase the pool of carbon that is potentially available for microorganisms, while its composition affects its metabolic fate in the microbial compartment. Coloured DOM was preferentially respired with little trophic transfer to the microbial food web, which may contribute to CO_2_ supersaturation and evasion to the atmosphere.

Our findings highlight terrestrial deliveries of chromophoric and humic DOM as a relevant component of the carbon cycle in headwater streams and thus complement recent findings from boreal^16^,^20^ and tropical^17^ systems. Upon entrance into streams, this DOM pool becomes subject to microbial degradation at the cost of low carbon use efficiency and potentially contributes to CO_2_ evasion to the atmosphere even without photochemical facilitation. Collectively, these findings contradict the common wisdom that “brown” DOM is resistant to microbial degradation and therefore largely exempt from metabolism en route from terrestrial sources to marine sinks^9^. Our findings may have important implications as terrigenous DOM deliveries into inland waters are predicted to increase and to cause browning^3^, especially where carbon loss from major stores such as peat and permafrost can be massive as climate change progresses.

## Methods

### Site description and sampling

We sampled twenty streams (1^st^-order, Austria) draining catchments with differing coverage of coniferous forest and peat (Table S1 and S2). Streamwater samples for DOC and optical analyses were filtered (Whatman GF/F filters) and stored in clean borosilicate vials. Triplicate streamwater samples were collected for the determination of *p*CO_2_ into 50-ml glass vials pre-conditioned with NaN_3_ (0.02% final concentration); at each site we also collected samples for atmospheric CO_2_. Streamwater for degradation assays was filtered as DOC samples and collected in clean polypropylene copolymer containers.

### Biodegradation assays

We conducted biodegradation assays to determine BDOC and CUE, and to relate them to DOM composition and to the observed shifts in DOM composition during microbial degradation. For each stream we conducted triplicate assays over 20 days (in the dark, 18°C). Sterile-filtered streamwater was inoculated with the native microbial community from the respective stream in 250 ml Schott-bottles with a headspace (38%). Samples for DOC concentration, DOM fluorescence and absorbance and CO_2_ were obtained at the start of the incubation and at day 3, 6, 10, 15 and 20, respectively. DOM samples were filtered and stored as described above. CO_2_ samples (10 ml) were collected from the headspace and injected into pre-evacuated exetainers. Abundance of microbial cells at each time point was determined using epifluorescence microscopy (Zeiss AxioImager) and microbial biomass was estimated from cell size and conversion factors. BDOC was calculated as the loss in DOC concentration over the 20-d incubation period. CUE was calculated as the increase in microbial biomass versus the increase in microbial biomass plus respiration (as dissolved inorganic carbon increase calculated from CO_2_ measurements over the same time period). Further details are provided in the Supplementary Information.

### DOM analyses

DOC concentration was measured using a total organic carbon analyzer (Sievers 5310C, GE, USA). DOM fluorescence and absorbance were determined using an Aqualog (Horiba, USA). Parallel factor analysis (PARAFAC) on excitation emission matrices^25^ resulted in three humic-like components but no protein-like component; this is plausible given the humic character of the streamwater (Figure 2). Components (C1, C2, C3) are expressed in percent relative to their total fluorescence. From fluorescence we further derived the humification index (HIX) indicative of the extent of humification^53^, the β/α index indicative of fresh microbially produced DOM^41^, and the fluorescence index (FI) as a proxy for DOM source (i.e., terrigenous *versus* microbially derived DOM)^42^. From absorption coefficients we derived the brown colour of DOM (*a*_440_)^54^, the specific UV absorption (SUVA_254_) as a proxy for aromaticity^55^, and the slope ratio (S_R_)^36^ and the ratio of absorption coefficients *a*_254_:*a*_365_^42^, which are both related to apparent DOM molecular weight. Further details are provided in the Supplementary Information.

### Streamwater ΔCO_2_ and respiration

CO_2_ concentration was measured using gas chromatography (Agilent) directly from the exetainers containing headspace samples from the degradation assays or after equilibration from the headspace (N_2_) in the streamwater samples. Anticipating shifts in carbonate fractions during sample equilibration we recalculated dissolved inorganic carbon (DIC) concentration using Henry's law and considering alkalinity and the respective equilibrium constants for the carbonate fractions (adjusted for temperature and ionic strength)^45^,^56^.We derived streamwater CO_2_ concentration (mol l^−1^) from DIC, pH and the equilibrium constants for the carbonate fractions^56^. The degree of CO_2_ supersaturation (expressed as ΔCO_2_ in μmol l^−1^) in the streams is the concentration of streamwater CO_2_ minus the CO_2_ concentration expected at complete atmospheric equilibrium; it indicates the CO_2_ evasion potential from the streams and serves as a proxy for heterotrophic metabolism. Further details are provided in the Supplementary Information.

### Statistical analyses

Principal component analysis (PCA) using optical measures normalized for DOC concentration served to explore DOM composition and to differentiate bulk DOM characterised by terrigenous or by autochthonous DOM contributions. Three separate canonical correlation analyses identified three linear sets of optical measures (as the respective first canonical axis) most strongly related to *a*_440_ (brown water colour), %BDOC and CUE as the constraints. Canonical loadings (i.e., correlations between the underlying variables and the one canonical axis) indicate the strength and the direction of the relationship of the individual DOM optical measure to the constraint. The strength of the relationship between the canonical axis and the constraint was computed as the canonical correlation coefficient, significance of each canonical correlation was computed by permutation.

## Author Contributions

C.F., G.A.S. and T.J.B. planned the research. C.F. and B.B. accomplished the fieldwork and the biodegradation experiments. C.F. conducted the CO_2_ measurements and all microbial work, B.B conducted the fluorescence spectrometry. C.F. performed the statistical analyses aided by G.A.S. T.J.B. wrote the manuscript with assistance from G.A.S., B.B. and C.F.

## Supplementary Material

Supplementary Information Supplementary Information
