# Supplementary material for: Microbial degradation of terrigenous dissolved organic matter and potential consequences for carbon cycling in brown-water streams
Source: Sci Rep. 2014 May 15;4:4981. doi: 10.1038/srep04981 (PMC4021337; doi:10.1038/srep04981)
Supplement: Supplementary Information [file srep04981-s1.pdf]

Supplementary Information for

Microbial degradation of terrigenous dissolved organic matter and possible consequences for carbon cycling in brown-water streams

Christina Fasching[1,]*, Barbara Behounek[1], Gabriel A. Singer[2,†] and Tom J. Battin[1, 2,] *

[1]Department of Limnology and Oceanography, University of Vienna, Althanstrasse 14, A-1090 Vienna, Austria

[2]WasserCluster Lunz GmbH, Dr. Carl Kupelwieser Promenade 5, 3293 Lunz am See

[†]Present address: Department of Ecohydrology, Leibniz-Institute of Freshwater Ecology and Inland Fisheries (IGB), Müggelseedamm 310, 12587 Berlin, Germany

* Corresponding authors: tom.battin@univie.ac.at and christinafasching@gmx.net

Methods

1. Degradation experiments

We conducted degradation experiments to determine bioavailable DOC (BDOC) and carbon use efficiency (CUE), and to relate these measures to DOM composition and to shifts in DOM composition during degradation as inferred from fluorescence and absorbance. For each stream we conducted triplicate degradation experiments over 20 days. We filled 180 ml sterile filtered streamwater into triplicate 250-ml pre-combusted (450°C, 4h) Schott© bottles and re-inoculated this water with unfiltered streamwater (10%) of the same stream, thereby leaving appr. 1/3 headspace. Bottles were incubated in the dark in a water bath at constant 18°C. After an equilibration period of 6 hours, during which bottles were loosely capped with combusted aluminum foil to avoid contamination but allow gas exchange, they were closed air-tight using screw caps and Teflon-coated septa (soaked with 10% $Na_2S_2O_8$ solution at 60°C for 1 h and rinsed thoroughly with MilliQ water). Samples for cell counts, DOC, optical analysis of DOM, and respiration (CO_2) were obtained at the beginning of the incubation period and after 1, 3, 6, 10, 15 and 20 days, respectively. Gas samples (i.e., 10 ml headspace of each bottle) were obtained with a gas tight syringe and transferred into 7-ml pre-evacuated headspace vials (exetainers), fitted with silicon sealed rubber stoppers and aluminum caps. Samples for DOC and optical analysis of DOM were filtered through a double layer of Whatman GF/F filters (precombusted, 450°C for 4h) into 40 ml glass vials (soaked with 0.1 N HCl, rinsed thoroughly with MilliQ water and combusted for 4h at 450°C); these were sealed with Teflon-coated septa (soaked with a 10% $Na_2S_2O_8$ solution at 60°C for 1 h and rinsed thoroughly with MilliQ water)

and stored at 4°C in the dark. Samples for cell counts (1 ml) were preserved in formaldehyde (2.5% final concentration) and stored at 4°C in the dark pending further processing.

Samples for cell counts were analysed using epifluorescence microscopy (Zeiss AxioImager) and the nucleic acid stain Sytox Green (Invitrogen). Stained cells were filtered onto black polycarbonate filters (Millipore) and 30 fields were photographed using AxioVision (4.6.1.0). We determined cell abundance, length and width for at least 200 cells per sample using image analysis (ImageJ 1.410). Image analysis was automated based on a size threshold (0.1 μm^2) identified by manually measured sizes. Cells with an area smaller than this threshold were excluded, as well as overlapping particles. We calculated cell volumes as $V = (w^2 * \pi/4) * (l - w) + (\pi * w^3/6)$ where V (μm^3) is the cell volume, and w and l are cell width and length (in µm). Employing the allometric relationship[1] $C = 120 * V^{0.72}$ we computed individual cell carbon content (fg). Microbial biomass was then calculated as the product of cell numbers and average cell carbon content for each sample. Bioavailable DOC (BDOC) was calculated as the change in DOC concentration over the 20-d incubation period. BDOC values were significantly related ($r^2 = 0.80$, $p < 0.001$, $n = 20$) to BDOC values derived from the sum of respiration and biomass (Figure S6). Carbon use efficiency (CUE) was computed as the increase in microbial biomass versus the increase in microbial biomass plus respiration (as DIC production, see below) over the identical time period (i.e., until stationary phase; day 15).

2. Analysis of DOC and optical properties of DOM

DOC samples from streamwater and the degradation experiments were analysed within 10 days of sampling using a Sievers 5310C TOC Analyzer operated with an inorganic carbon removal unit. Prior to injection, DOC samples were automatically acidified in the analyser as recommended by the manufacturer. The method detection limit of the Sievers 5310C according to US EPA guidelines[2] was typically below 6 $\mu g\ C\ l^{-1}$.

Samples for optical analysis of DOM were measured within 14 days of sampling. Absorbance scans and excitation emission matrices (EEMs) were generated simultaneously using an Aqualog® (Horriba Scientific). Fluorescence intensities were measured at excitation wavelengths ranging from 250 to 450 nm (5-nm increments) and emission wavelengths from 250 to 550nm (2-nm increments). The water Raman peak of Milli-Q water served as reference. EEMs were corrected for blanks (MilliQ) and absorbance (inner filter effect). Using parallel factor analysis (PARAFAC) [3], we modeled individual fluorescent components from the obtained EEMs. Modeling was performed in Matlab (7.11.0) using the DOMFluor Toolbox (1.7; containing the N-Way toolbox, 3.1)[4]. PARAFAC identified three humic-like components but no protein-like component, which is plausible given the humic character of the water (Figure 2). Component 1 (Em 455 nm / Ex < 250 (335) nm) and 2 (Em 500 nm / Ex < 250 (400 nm) resembled the classical peaks C and A, respectively, as described by Coble[5]. Both peaks are of terrestrial origin and commonly found in freshwater environments[6]. In contrast, component 3 (Em 408

nm/ Ex < 250 (305) nm) is putatively associated with biological production *in situ* and therefore diagenetically younger[7].

To characterize DOM properties and composition we computed the following indices from DOM absorbance and fluorescence: The humification index (HIX) was calculated as the peak area of the emission wavelengths from 435 to 480 nm divided by the peak area of the emission wavelengths from 300 to 445 nm, at an excitation wavelength of 254 nm[8]. Higher values indicate higher humic substance content or extent of humification[8].The ß/α index was computed as the ratio of emission intensity at 380 nm (ß) to the maximum emission intensity between 420 and 435 nm (α) at an excitation wavelength of 310 nm[9]. High ß/α values indicate autochthonous inputs, i.e., recently microbially produced DOM[10]. The fluorescence index (FI) was calculated as the ratio of emission intensity at 450 nm to that at 500 nm for an excitation wavelength of 370 nm[11]; it is commonly used as an indicator of DOM source (i.e. terrigenous vs. microbially derived DOM)[11]. The slope ratio (S_R) was calculated as the ratio of $S_{275-295}$ to $S_{350-400}$; it is reported to correlate inversely with DOM molecular weight[12]. The specific UV absorption ($SUVA_{254}$) was calculated as the absorption coefficient at 254 nm (m^{-1}) relative to the DOC concentration (mg l^{-1})[13]. The $SUVA_{254}$ was reported to correlate positively with increasing DOM aromaticity[13]. Additionally we calculated the ratio of absorbance at 254 and 365nm (a254/a365), which was shown to be negatively correlated to molecular weight of DOM[14, 15].

3. Nutrients, ions and field parameters

Concentrations of $N-NH_4$, $N-NO_2$, $N-NO_3$, $P-PO_4$ in the streamwater were determined using Continuous Flow Analysis (Alliance instruments). Detection limits for nutrient analyses were 4 µg l^{-1} for $N-NH_4$, 100 µg l^{-1} for N-NO3 and 2 µg l^{-1} for $P-PO_4$. Ion concentrations (Na^+, K^+, Ca^{2+}, Mg^{2+}, Cl^-, NO_3, and SO_4^{2-}) were measured using ion chromatography (761 Compact IC; Methrom). Water temperature and pH were measured using regularly calibrated WTW probes (Cond340i, pH320, Weilheim -Germany) and a calibrated high-precision glass electrode for pH (Metrohm), respectively. Alkalinity was determined by Titration with 0.1 mM HCl.

4. Streamwater ΔCO_2 and respiration

To determine the streamwater CO_2 concentration we carefully submersed triplicate 50-ml glass vials (pre-conditioned with precipitated NaN_3, 0.02 % final concentration) into the streamwater, and allowed them to overflow before closing them under water with a 1 cm thick gas-tight rubber stopper. While closing the vials a needle was inserted into the rubber stopper to avoid disturbance and allow excess water to emerge from the vial. Triplicate atmospheric samples (10 ml each) were obtained using a gas-tight syringe and injected into pre-evacuated 7-ml headspace vials, fitted with silicon sealed rubber stoppers and aluminum caps. Gas samples were stored at 4°C in the dark for a maximum

of 2 days. Prior to measurement a headspace of known volume was generated with N_2 in the liquid samples. Equilibration of the gaseous and the liquid phase was then achieved by means of a water bath at controlled temperature aided by shaking for 45 min. Samples were analysed using gas chromatography (Agilent Technologies 7890A) and a headspace autosampler. Temperature was kept constant during measurement.

During sample equilibration at lab conditions, CO_2 evades from the liquid phase into the N_2-headspace, causing the equilibria of the carbonate fractions (CO_2, HCO_3^- and CO_3^{2-}) to shift. Thus, the CO_2 concentration measured in the headspace cannot be directly related to the CO_2 concentration in the liquid phase in the stream and at the respective field conditions. We accounted for this by calculating the total DIC concentration of the sample assuming constant alkalinity and employing the equilibrium constants for the respective carbonate fractions[16, 17] and respective temperature and pressure conditions. Briefly, we computed the amount of moles of CO_2 in the liquid phase from measured CO_2 partial pressure in the headspace and Henry´s law assuming full equilibration. This computed sample CO_2 concentration (mol l^{-1}), alkalinity and the respective equilibrium constants for the carbonate fractions (adjusted for temperature and ionic strength) were used to infer pH. Then the concentration of HCO_3^- and CO_3^{2-} could be calculated using pH and the adequate equilibration constants (adjusted for temperature and ionic strength). The sum of all carbonate fractions (in moles) in the liquid phase and the total amount of CO_2 (in moles) in the headspace gave the total amount of DIC (in moles).

We then calculated the CO_2 concentration (mol l^{-1}) in the streamwater from computed DIC values and measured pH values using the equilibrium constants (adjusted for temperature and ionic strength). A potential streamwater CO_2 concentration in mol l^{-1} at equilibrated conditions was calculated from the measured atmospheric CO_2 concentrations using Henry´s law. The difference of the CO_2 concentration in the stream in mol l^{-1} to the concentration expected from the atmospheric equilibrium gives the degree of CO_2 oversaturation (ΔCO_2).

During the degradation experiments CO_2 production causes the equilibrium of the carbonate fractions (CO_2, HCO_3^- and CO_3^{2-}) to shift. Thus, we used the same calculation scheme as for the liquid samples to recalculate total DIC concentrations. From measured pCO_2 concentrations we derived the amount of moles of CO_2 in the liquid phase using Henry´s law. Employing the equilibrium constants for the respective carbonate fractions and alkalinity (constant and measured at the start of the degradation experiments) we recalculated a pH value for each time point [Dickson et al., 2007; Berggren *et al.*, 2012]. Taking into account the pH values and the equilibrium constants for the respective carbonate fractions we computed all carbonate fractions. The sum of headspace CO_2 concentration (in moles) and the carbonate fractions (in moles) gave the total DIC concentration for each incubation time point.

Table S1 Geographical location, dissolved organic carbon (DOC) concentration, bioavailability (BDOC), color (a_{440}) and carbon use efficiency (CUE) of all 20 streams. Longitude is given in decimal degrees East and latitude in decimal degrees North.

Stream	Longitude (°E)	Latitude (°N)	DOC (mgl^{-1})	a440 (m^{-1})	BDOC (%)	CUE (%)
A	15.0075	48.3736	9.21 ± 0.04	7.04 ± 0.4	3.62	5.77
B	15.0028	48.3586	5.25 ± 0.04	4.02 ± 0.02	4.11	6.01
C	14.9853	48.3111	1.41 ± 0.02	0.78 ± 0.06	8.18	9.55
D	14.9675	48.3547	7.63 ± 0.02	6.08 ± 0.1	5.04	8.97
E	14.9633	48.3694	2.66 ± 0.01	1.90 ± 0.02	9.30	5.31
F	14.9656	48.3786	5.31 ± 0.06	4.32 ± 0.1	8.18	9.04
G	15.1053	48.3842	5.16 ± 0.05	3.49 ± 0.17	4.67	7.81
H	15.0808	48.3792	19.49 ± 0.23	17.52 ± 0.03	4.25	4.57
I	15.0450	48.4086	6.85 ± 0.04	3.59 ± 0.06	1.63	15.24
J	15.0314	48.4028	10.04 ± 0.05	7.36 ± 0.1	4.04	10.85
K	15.0439	48.3975	3.28 ± 0.03	1.96 ± 0.08	4.07	6.36
L	15.0436	48.3806	10.60 ± 0.02	8.00 ± 0.05	3.72	11.39
M	14.8722	48.5047	6.07 ± 0.04	4.15 ± 0.07	3.33	20.63
N	14.8619	48.5047	25.31 ± 0.07	26.64 ± 0.19	5.09	5.07
O	14.8533	48.5222	7.48 ± 0.12	5.09 ± 0.17	4.26	8.00
P	14.8367	48.5594	4.33 ± 0.12	2.09 ± 0.02	7.06	9.91
Q	14.7619	48.5961	2.48 ± 0.01	1.26 ± 0.06	4.38	37.10
R	14.7306	48.5969	4.50 ± 0.01	2.33 ± 0.01	2.33	11.58
S	14.7069	48.5564	7.02 ± 0.02	3.73 ± 0.14	1.87	27.86
T	14.6861	48.5511	5.42 ± 0.05	3.25 ± 0.16	1.92	33.65

Table S2 PH, electrical conductivity and nutrients (NH_4, NO_2, NO_3, PO_4) of all 20 streams.

			Nutrients at the start of the biodegradation assays				Nutrients at the end of the biodegradation assays			
Stream	pH	Conductivity ($\mu S\ cm^{-1}$)	NH_4 ($\mu g\ l^{-1}$)	NO_2 ($\mu g\ l^{-1}$)	NO_3 ($\mu g\ l^{-1}$)	PO_4 ($\mu g\ l^{-1}$)	NH_4 ($\mu g\ l^{-1}$)	NO_2 ($\mu g\ l^{-1}$)	NO_3 ($\mu g\ l^{-1}$)	PO_4 ($\mu g\ l^{-1}$)
A	6.69	37.60	5.33 ± 2.03	3.37 ± 0.35	301.77 ± 3.96	21.37 ± 0.71	6.33 ± 3.73	3.20 ± 0.10	302.53 ± 8.37	20.43 ± 1.15
B	6.92	49.20	< 4	2.60 ± 0.44	787.70 ± 3.04	16.30 ± 0.35	5.60 ± 4.55	2.33 ± 0.40	801.37 ± 20.05	15.63 ± 0.06
C	7.30	81.50	< 4	1.00 ± 0.36	1414.93 ± 9.00	13.17 ± 0.68	7.40 ± 2.19	1.13 ± 0.15	1427.27 ± 4.91	14.53 ± 0.64
D	6.81	44.70	4.43 ± 1.82	4.03 ± 0.23	451.40 ± 2.74	17.90 ± 0.35	5.63 ± 1.58	2.83 ± 0.06	457.40 ± 6.68	17.37 ± 0.32
E	7.00	71.40	4.35 ± 1.34	1.37 ± 1.12	662.23 ± 10.84	4.10 ± 0.00	13.03 ± 3.42	1.30 ± 0.61	665.33 ± 8.20	5.30 ± 0.36
F	7.02	56.10	6.30 ± 4.24	6.23 ± 0.75	534.17 ± 17.47	5.75 ± 0.78	15.70 ± 3.45	2.60 ± 0.26	534.13 ± 7.97	6.13 ± 0.12
G	6.83	59.60	< 4	2.37 ± 0.25	515.77 ± 5.80	21.60 ± 3.00	< 4	2.20 ± 0.53	503.80 ± 6.90	18.07 ± 0.78
H	6.36	43.00	< 4	1.47 ± 0.67	519.10 ± 0.85	10.80 ± 0.95	14.73 ± 1.46	9.27 ± 0.15	< 100	54.87 ± 0.85
I	6.84	51.30	< 4	< 1	714.40 ± 4.83	9.47 ± 0.40	< 4	< 1	155.23 ± 4.75	9.90 ± 0.95
J	7.14	72.40	12.27 ± 0.25	8.93 ± 0.23	< 100	59.37 ± 0.59	4.57 ± 1.27	3.57 ± 0.21	690.60 ± 5.80	14.20 ± 0.53
K	6.77	67.50	7.30 ± 1.65	5.27 ± 0.61	700.10 ± 3.58	14.73 ± 0.40	4.27 ± 3.57	1.23 ± 0.12	722.93 ± 8.46	11.23 ± 1.55
L	6.90	59.40	6.07 ± 0.78	5.60 ± 0.66	596.13 ± 3.44	21.70 ± 0.26	9.70 ± 3.08	3.87 ± 0.31	596.33 ± 4.01	19.20 ± 0.10
M	6.88	53.00	< 4	3.57 ± 0.31	571.93 ± 3.18	28.63 ± 2.17	7.77 ± 1.19	3.07 ± 0.12	567.40 ± 3.99	39.80 ± 1.30
N	6.53	55.40	251.40 ± 1.14	16.80 ± 0.44	267.43 ± 0.91	102.23 ± 2.72	19.53 ± 1.80	n.d.	n.d.	77.17 ± 5.15
O	7.14	76.30	7.80 ± 0.96	2.20 ± 0.50	373.00 ± 1.21	48.05 ± 0.35	7.47 ± 3.93	3.20 ± 0.50	368.70 ± 4.88	32.77 ± 1.88
P	7.16	122.50	4.73 ± 0.25	1.13 ± 0.21	649.70 ± 1.71	13.60 ± 0.71	< 4	1.80 ± 0.30	626.77 ± 3.09	14.17 ± 1.06
Q	7.40	110.70	< 4	1.33 ± 0.51	592.50 ± 5.41	11.67 ± 0.23	4.97 ± 1.70	< 1	591.70 ± 8.41	12.67 ± 0.93
R	6.75	62.20	4.57 ± 1.15	1.07 ± 0.45	146.17 ± 2.51	6.65 ± 0.64	8.40 ± 1.14	1.10 ± 0.53	155.83 ± 4.72	8.33 ± 0.59
S	6.93	64.40	4.23 ± 1.39	1.80 ± 0.20	232.60 ± 2.95	9.50 ± 0.46	5.73 ± 2.34	1.97 ± 0.46	253.30 ± 2.27	11.70 ± 0.98
T	6.81	131.60	< 4	2.37 ± 0.15	283.07 ± 2.37	9.50 ± 1.08	4.87 ± 2.27	1.97 ± 0.12	295.87 ± 3.36	10.73 ± 1.30

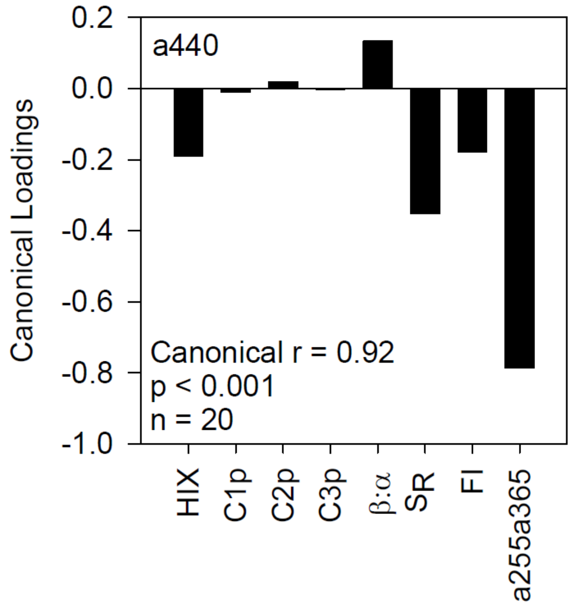

Figure S1 Canonical correlation analysis based on the optical information of the 20 streams reveals the DOM optical properties which are most strongly related to absorbance at 440 nm (a_{440}). This analysis is identical to the one presented in Figure 3 but excluding specific absorbance at 440 nm (SA_{440}) and specific UV absorbance at 254 nm($SUVA_{254}$) in order to test for any spurious effect of DOM quantity. Canonical loadings (correlations between the one canonical variate of our analysis and the respective variables) indicate the strength and direction of the relationship between the constraint (a_{440}) and individual optical measures: component 1 (C1), component 2 (C2), component 3 (C3), humification index (HIX), fluorescence index (FI), freshness index (ß/α), slope ratio (S_R), the ratio of absorbance at 254 to 365nm (*a*254/*a*365). Despite the exclusion of the concentration-dependent parameters $SUVA_{254}$ and SA_{440} canonical loadings indicate an increase of the terrigenous signal with a_{440}.

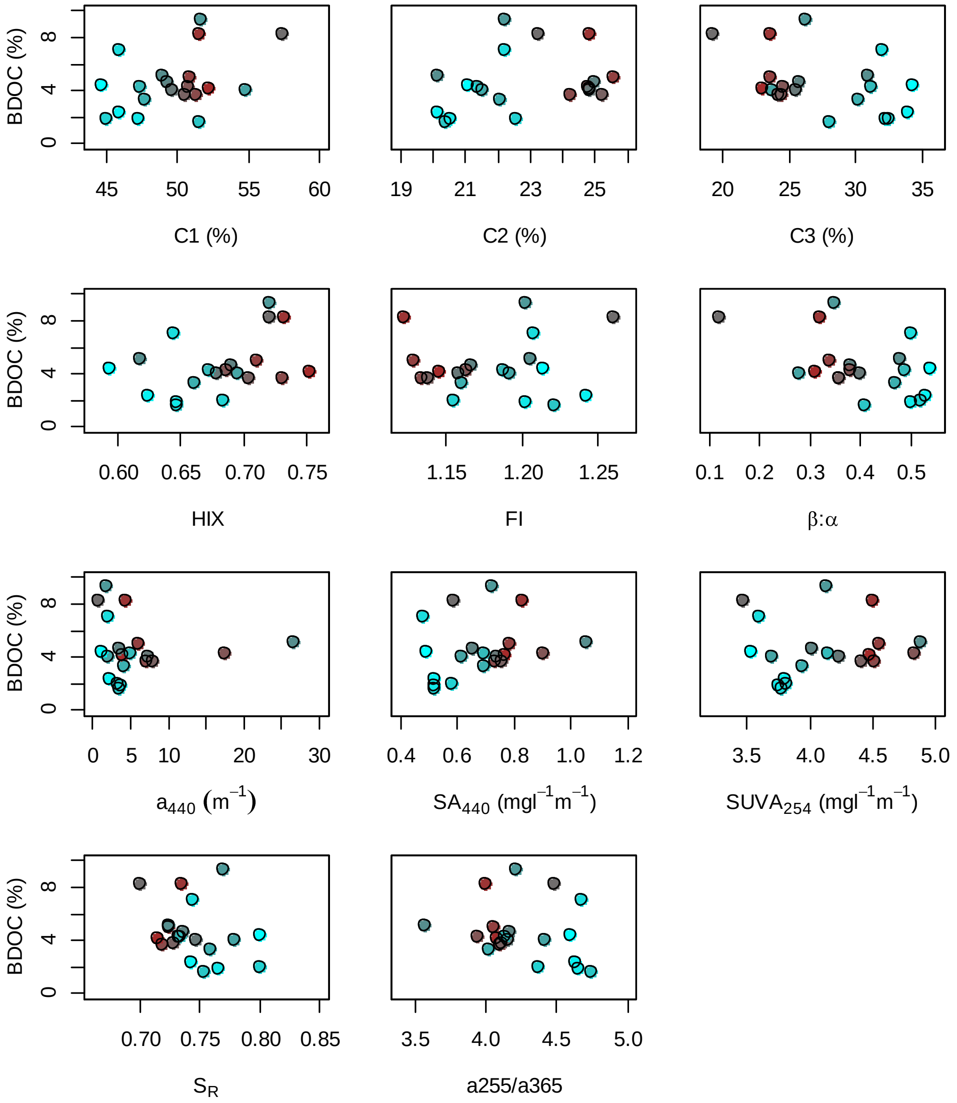

Figure S2 Relationships of bioavailable DOC (%BDOC) with various optical measures: component 1 (C1), component 2 (C2), component 3 (C3), humification index (HIX), fluorescence index (FI), freshness index (ß/α), absorbance at 440 nm (*a*440), specific (i.e., normalized for DOC) absorbance at 440 nm (SA_{440}), specific UV absorbance at 254 nm ($SUVA_{254}$), slope ratio (S_R), and the ratio of absorbance at 254 to 365 nm (*a*254/*a*365). Original DOM quality as derived from the PCA is indicated by colour. Brown colors denote streams with predominantly terrigenous DOM and blue colors represent streams with relatively more autochthonous DOM.

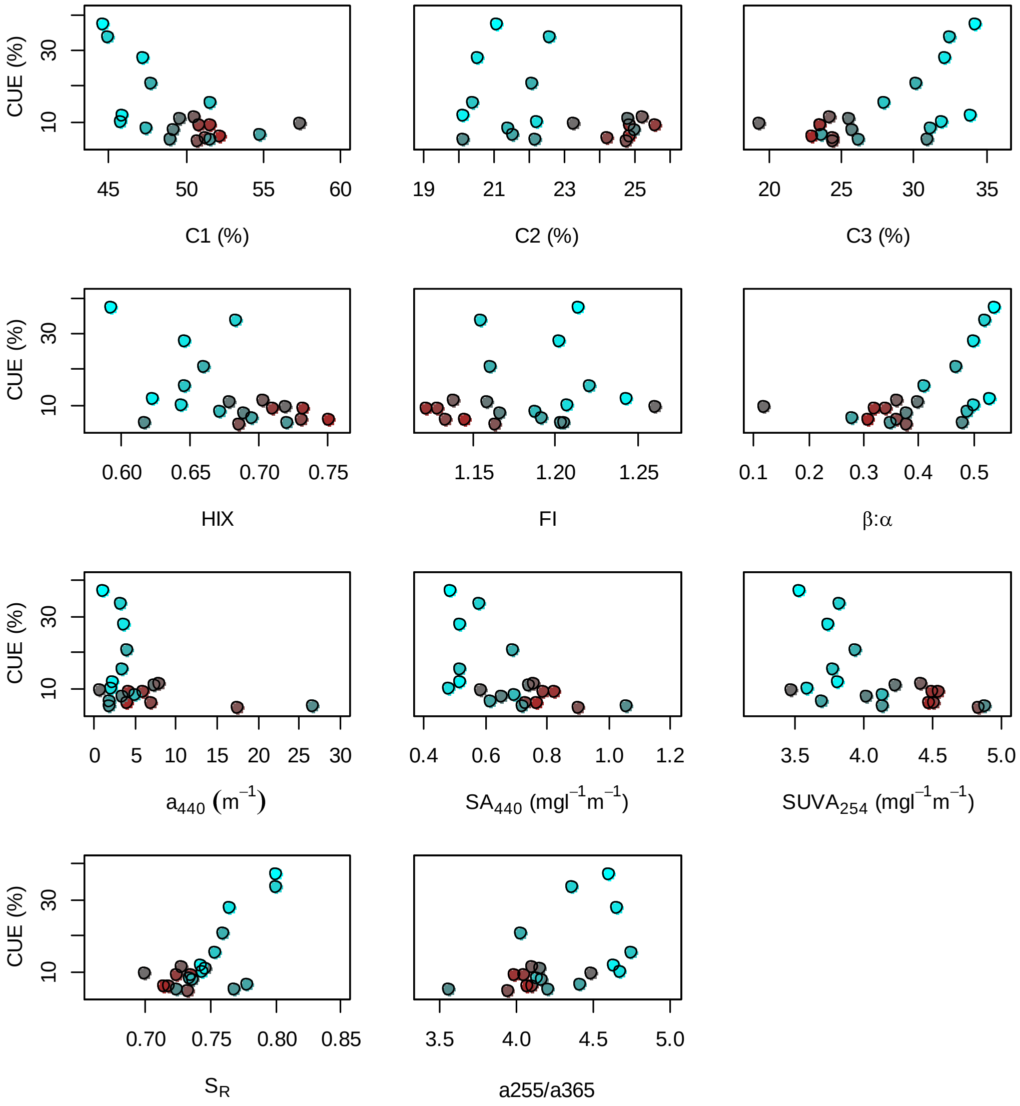

Figure S3 Relationships of carbon use efficiency (CUE) and various optical measures: component 1 (C1), component 2 (C2), component 3 (C3), humification index (HIX), fluorescence index (FI), freshness index (ß/α), absorbance at 440 nm (*a*440), specific (i.e., normalized for DOC) absorbance at 440 nm (SA_{440}), specific UV absorbance at 254 nm ($SUVA_{254}$), slope ratio (S_R), and the ratio of absorbance at 254 to 365 nm (*a*254/*a*365). Original DOM quality as derived from the PCA is indicated by colour Brown colors denote streams with predominantly terrigenous DOM and blue colors represent streams with relatively more autochthonous DOM.

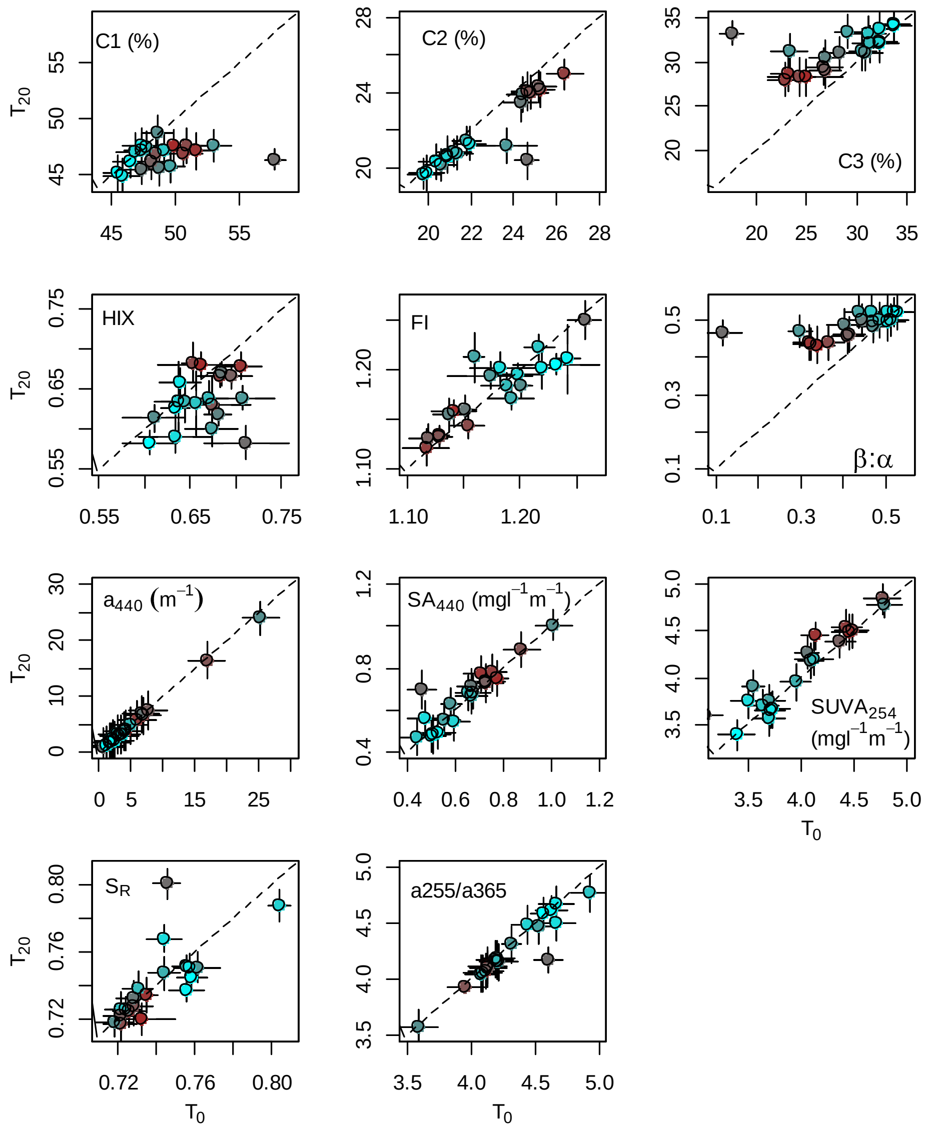

Figure S4 Optical measures at the start of the degradation experiments (T0) and after the 20-day incubation period (T20): component 1 (C1), component 2 (C2), component 3 (C3), humification index (HIX), fluorescence index (FI), freshness index (ß/α), absorbance at 440 nm (*a*440), specific (i.e., normalized for DOC) absorbance at 440 nm (SA_{440}), specific UV absorbance at 254 nm ($SUVA_{254}$), slope ratio (S_R), and the ratio of absorbance at 254 to 365 nm (*a*254/*a*365). Given is the mean ± SD (n=3 replicates) and the dashed line indicates a 1:1 relationship (i.e., no change during degradation) Original DOM quality as derived from the PCA is indicated by colour. Brown colors denote streams with predominantly terrigenous DOM and blue colors represent streams with relatively more autochthonous DOM.

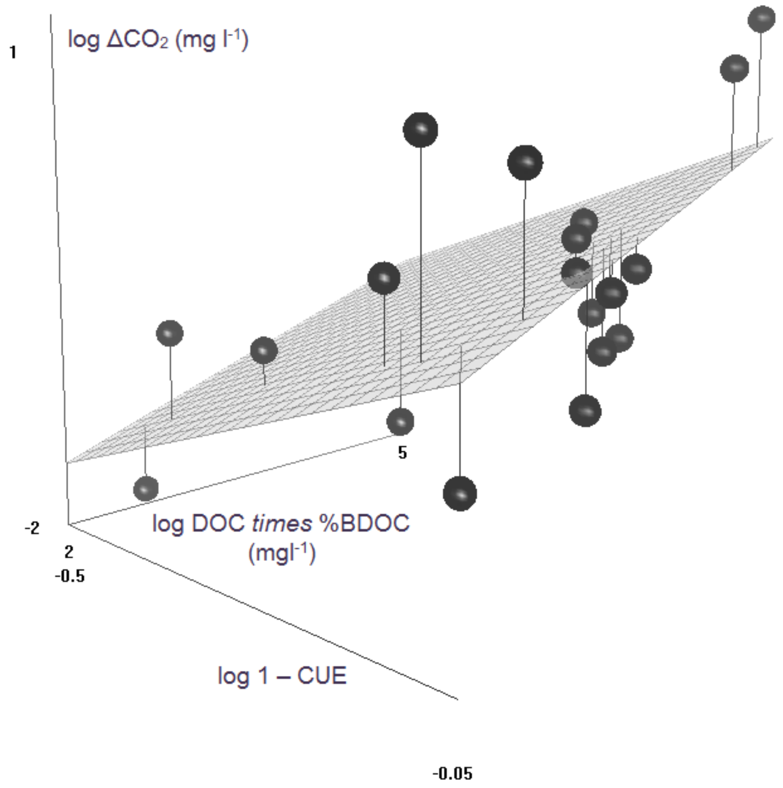

Figure S5 Bioavailable dissolved organic carbon and carbon use efficiency predict streamwater CO_2 partial pressure. Symbols indicate measured values with lines (residuals) connected to the modeled surface.

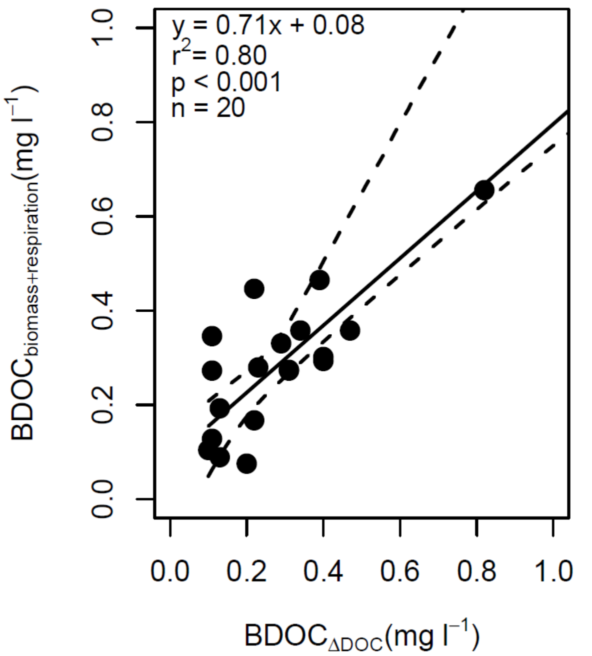

Figure S6 Ranged major axis regression of the BDOC values measured as the decrease in DOC concentration over the incubation period ($BDOC_{\Delta DOC}$) and BDOC values derived from the sum of respiration and biomass. Significance was computed by permutation and dashed lines give the 95% model confidence level.

References

1. Norland, S. The relationship between biomass and volume of bacteria. In: Kemp, P.F., Sherr, B., Cole, J.J. (eds) Handbook of methods of aquatic microbial ecology. Lewis, Boca Raton, pp 303–308 (1993).
2. US EPA. Code of Federal Regulations, Title 40 - Protection of Environment, Chapter 1 - Environmental Protection Agency, Subchapter D - Water Programs, Part 136 - Guidelines establishing test procedures for the analysis of pollutants, Appendix B - Definition and Procedure for the Determination of the Method Detection Limit.
3. Stedmon, C. A. and Bro, R. Characterizing dissolved organic matter fluorescence with parallel factor analysis: a tutorial. *Limnol. Oceanogr. Meth.* **6**, 572–579 (2008).
4. Andersson, C. A. and Bro, R. The N-way Toolbox for MATLAB. *Chemometrics Intell. Lab. Syst.* **52**, 1-4 (2000).
5. Coble, P.G. Characterization of marine and terrestrial OM in seawater using excitation emission matrix spectroscopy. *Mar. Chem.* **51**, 325–346 (1996).
6. Stedmon, C .A., Markager , S. and Bro, R. Tracing dissolved organic matter in aquatic environments using a new approach to fluorescence spectroscopy. *Mar. Chem.* **82**, 239-254 (2003).
7. Cory, R. M and Kaplan, L. A. Biological lability of streamwater fluorescent dissolved organic matter. *Limnol. Oceanogr.* **57**, 1347–1360 (2012).
8. Zsolnay, A., Baigar, E., Jimenez, M., Steinweg, B., Saccomandi F. Differentiating with fluorescence spectroscopy the sources of dissolved organic matter in soils subjected to drying, *Chemosphere* **38**, 45-50 (1999).
9. Parlanti, E., Wortz, K., Geoffroy, L. and Lamotte, M. Dissolved organic matter fluorescence spectroscopy as a tool to estimate biological activity in a coastal zone submitted to anthropogenic inputs. *Org. Geochem.* **31**, 1765–1781 (2000).
10. Wilson, H.F. and Xenopoulos, M. A. Effects of agricultural land use on the composition of fluvial dissolved organic matter. *Nature Geosci.* **2**, 37 – 41 (2009).
11. McKnight, D.M., Boyer, E. W., Westerhoff, P. K., Doran, P. T., Kulbe, T., Andersen, D. T. Spectrofluorometric Characterization of Dissolved Organic Matter for Indication of Precursor Organic Material and Aromaticity. *Limnol. Oceanogr.* **46**, 38 – 48 (2001).

12. Helms, J. R., Stubbins, A., Ritchie, J. D., Minor , E. C., Kieber, D. J., Mopper K. Absorption spectral slopes and slope ratios as indicators of molecular weight, source, and photobleaching of chromophoric dissolved organic matter. *Limnol. Oceanogr.* **53**, 955-969 (2008).
13. Weishaar, J.L., Aiken, G. R., Bergmaschi, B. A., Fram, M. S., Fugii R. and Mopper, K., Evaluation of specific ultraviolet absorbance as an indicator of the chemical composition and reactivity of dissolved organic carbon. *Environ. Sci. Technol.* **37**, 4702-4708 (2003).
14. De Haan, H. Solar UV-Light Penetration and Photodegradation of Humic Substances in Peaty Lake Water. *Limnol. Oceanogr.* **38**, 1072-1076 (1993).
15. Berggren, M., Laudon, H. and Jansson, M. Landscape regulation of bacterial growth efficiency in boreal freshwaters, *Global Biogeochem. Cycles* 21, GB4002, doi:10.1029/2006GB002844 (2007).
16. Dickson, A.G., Sabine, C. L. and Christian, J. R. (Eds.). Guide to best practices for ocean CO2 measurements. PICES Special Publication **3**, 191 pp (2007).
17. Berggren, M., Lapierre, J. –F. and del Giorgio, P. A. Magnitude and regulation of bacterioplankton respiratory quotient across freshwater environmental gradients. *ISME J.* **6**, 984–993 (2012).
